# Incidence of pedicle breach following open and minimally invasive spinal instrumentation: A postoperative CT analysis of 513 pedicle screws applied under fluoroscopic guidance

**DOI:** 10.37796/2211-8039.1016

**Published:** 2020-06-05

**Authors:** Xue Ling Chong, Aravind Kumar, Eugene Wei Ren Yang, Arun-Kumar Kaliya-Perumal, Jacob Yoong-Leong Oh

**Affiliations:** a Division of Spine, Department of Orthopaedic Surgery, Tan Tock Seng Hospital, Singapore; b Department of Orthopaedic Surgery, Ng Teng Fong General Hospital, Singapore; c Department of Neurosurgery, Khoo Teck Puat Hospital, Singapore

**Keywords:** Fluoroscopy, Pedicle Screws, Spine, Spinal Fractures, Vertebral column

## Abstract

**Background:**

Even though pedicle screw application is a common procedure, and in-spite of spine surgeons being proficient with the technique, mal-positioning of pedicle screws can still occur. We intend to determine by postoperative CT analysis, the incidence of pedicle screw breach in the thoracolumbar spine despite satisfactory intraoperative placement confirmed by fluoroscopy.

**Materials and methods:**

Consecutive patients diagnosed with thoracolumbar fractures who underwent open or minimally invasive posterior stabilization under fluoroscopic guidance were retrospectively reviewed. Postoperative CT scans of patients were analysed to determine the incidence of pedicle breach despite satisfactory intraoperative placement, and also to determine the factors that can predict a breach during intraoperative assessment.

**Results:**

A total of 61 patients with 513 thoracolumbar pedicle screws were available for analysis. Based on our postoperative CT assessment, 28 screws (5.5%; 18 thoracic screws; 10 lumbar screws) had breached the pedicle. There were 14 minor (<3 mm) and 14 major (≥3 mm) breaches. The minimally invasive technique had a significantly lower breach rate compared to open surgery (1.9% vs. 7.9%). By retrospectively analysing the intra-operative fluoroscopic images, we determined certain parameters that could predict a breach during surgery.

**Conclusion:**

Pedicle breaches can still be present despite satisfactory placement of screws visualized intra-operatively. A medial breach is most likely when the length of the pedicle screw spans only up to 50% of the vertebral body as seen on the lateral view but the pedicle screw tip has already transgressed the midline as seen on an AP view. A lateral breach is likely when the tip of the pedicle screw is overlapped by the screw head or is only minimally visualized on an AP view.

## 1. Introduction

Posterior stabilization using pedicle screws and rods is a long-established gold standard technique of spinal fixation which plays an important role during surgery for spinal instabilities, deformity, degeneration, infection and tumours [1–7]. Although it has become a common procedure, and in-spite of spine surgeons being proficient with the technique, malpositioning of pedicle screws can still occur [8, 9]. Such malpositioning not only compromises the stability of the construct but also endangers neural integrity [10]. Various authors have reported different rates of pedicle breach ranging between 1.7% to 35% during open pedicle screw placement and 2.6% to 12.3% during minimally invasive pedicle screw placement [11–14]. For both open and minimally invasive techniques, intraoperative assessment of pedicle breach is generally done with the help of fluoroscopy; however, such interpretation may be affected by the screw length, rotation and image quality. Therefore, we would like to determine by postoperative CT analysis, the incidence of pedicle screw breach despite normal intraoperative imaging with fluoroscopy. We also performed a retrospective review of those patients who had a pedicle breach to determine the errors that may have caused the surgeon to accept that the screws were perfect by assessing the fluoroscopic image.

## 2. Materials and methods

With approval from our institutional review board, electronic records of consecutive patients diagnosed with thoracolumbar fractures who underwent posterior stabilization during a particular period were retrospectively reviewed. Only those patients who had their intraoperative fluoroscopic images and postoperative CT scan images saved in our picture archiving and communication system (PACS) were included for analysis. Since we do not routinely perform a postoperative CT scan except for patients with thoracolumbar fractures, other patients with tumour, infection, degenerative conditions, and deformity, had to be excluded. All procedures were performed by the same surgical team using either the open approach or the minimally invasive approach, and the pedicle screws were placed under fluoroscopic guidance.

In the open approach, a standard posterior midline incision was made, and layer wise dissection was carried out to expose the posterior elements including the facet joints and transverse process of the segments to be instrumented. The entry point for both the thoracic and lumbar pedicles were made as per described techniques based on anatomical landmarks [15, 16]. Pedicle probing and tapping were done free hand, and the trajectory was confirmed using fluoroscopy, followed by pedicle screw insertion. In the minimally invasive approach, either multiple stab incisions were made on the skin or a single midline skin incision with multiple stab incisions on the facia was made. Unlike the open approach, the entry point was made under fluoroscopic guidance with a Jamshidi needle through which a guide wire was inserted. Tapping was carried out after removal of the needle leaving the guide wire in place which was later removed after placement of a cannulated pedicle screw. It was mandatory that all the pedicle screws demonstrated acceptable placement in the intraoperative fluoroscopic image and if not, the screws were revised until satisfactory placement was achieved.

The postoperative CT scans were analysed by a clinician who was not a part of the surgical team. Pedicle breach was classified into three grades: 1) No breach, 2) Minor breach (<3 mm) and 3) Major breach (≥3 mm). This classification was based on other published clinical studies demonstrating that breach rates up to 3 mm could be associated without serious complications [17]. We also performed a subgroup analysis comparing breach rates between open and minimally invasive techniques. The intra-operative fluoroscopic images of those patients identified with pedicle breach post-operatively were analysed to determine the factors that may have influenced the surgeon to accept that the screws were perfect during the intraoperative assessment.

All statistical analysis was performed using Stata, version 13 (StataCorp LP, TX), with statistical significance being defined as a p value of <0.05. The Chi-square test or Fischer's exact test was used wherever appropriate to determine significance. The study was approved by our institutional review board and was performed in accordance with the ethical standards laid down in the most recent version of the 1964 Declaration of Helsinki, or comparable ethical standards.

## 3. Results

**Fig. 1:**
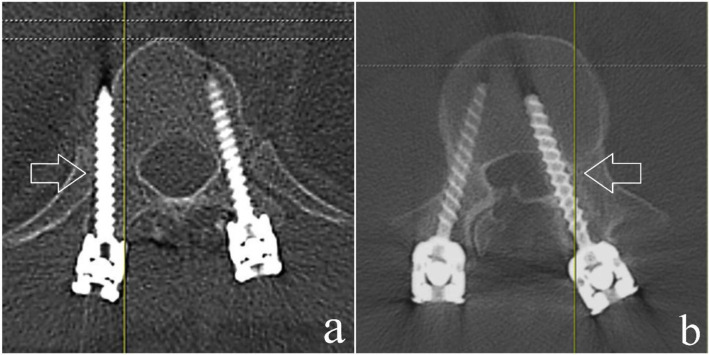
Post-operative axial CT image depicting a) Minor breach (<3 mm) and b) Major breach (≥3 mm).

**Fig. 2:**
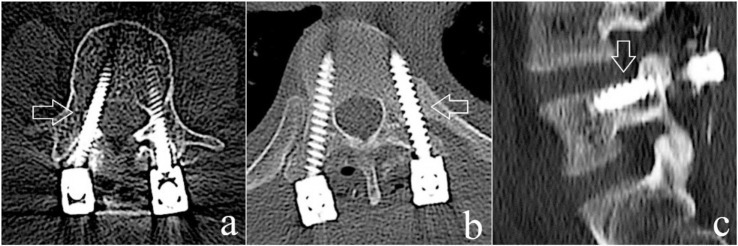
Postoperative CT image showing various directions of pedicle screw breach. a) Medial; b) Lateral; c) Superior.

The selected sample consisted of 61 patients (Male = 40; Female = 21) with a mean age of 40 years (Range = 19 – 77 years). All patients had sustained thoracolumbar injuries and underwent surgery in the form of posterior stabilization using pedicle screws and rods. A total of 513 pedicle screws were applied under fluoroscopic guidance. Based on our post-operative CT assessment, 28 screws (5.5%) had pedicle breaches; among which, 18 occurred in the thoracic spine, while 10 occurred in the lumbar spine (Table 1). There were 14 major and 14 minor breaches, constituting for 2.75% each. 17 (61%) of the breaches were medial and 10 (36%) were lateral (Fig. 1). There was only 1 superior breach and no inferior breach (Fig. 2). Based on our subgroup analysis, minimally invasive screw fixation (29 patients; 210 screws) had a breach rate of 1.9% (4 screws) as compared to open fixation (32 patients; 303 screws) which had a breach rate of 7.9% (24 screws). This difference was clinically significant (p = 0.0048). Despite the pedicle breach, none of our patients had any neurological compromise.

**Fig. 3:**
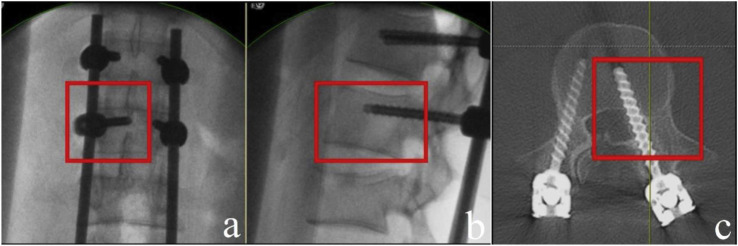
a) Intra-operative fluoroscopy image showing the left lumbar level 2 pedicle screw tip touching the midline on a PA view; b) Screw length spanning up to 50% of the vertebral body in the lateral view; c) Post-operative axial CT image showing a major medial breach of the pedicle.

**Fig. 4:**
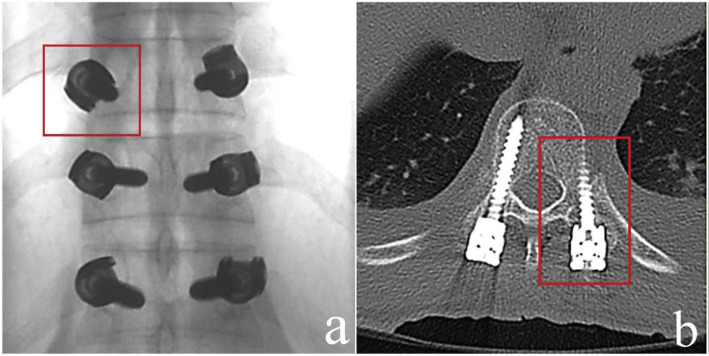
a) Intra-operative fluoroscopy image showing the tip of the left thoracic level 10 pedicle screw being overlapped by the screw head; b) Postoperative axial CT image showing a major lateral beach of the pedicle.

By retrospectively evaluating the intra-operative radiographs of patients who were identified to have a breach, we derived some fluoroscopic parameters that would suggest the same during surgery. A medial breach is most likely when the length of the pedicle screw spans only up to 50% of the vertebral body as seen on the lateral view but the pedicle screw tip has already transgressed the midline as seen on an AP view (Fig. 3). A lateral breach is likely when the tip of the pedicle screw is overlapped by the screw head or is only minimally visualized on an AP view (Fig. 4).

## 4. Discussion

Technological advancements have made spine surgery safer, predictable and precise [18–20]. However, recent advancements such as computer navigation and robotic assistance for placement of pedicle screws come at a cost and are not universally available at present [18]. Hence, surgeons at most institutions rely on conventional intra-operative fluoroscopy to place pedicle screws, a technique that is long proven to be effective [21]. Despite satisfactory placement of the pedicle screws as seen on intra-operative fluoroscopic imaging, pedicle breaches can still be present [8, 9]. This could be due to false interpretation influenced by the screw length, rotation and image quality. These factors can affect the assessment of screw placement both during open and minimally invasive procedures. Literature has produced mixed data on whether the percutaneous approach has shown better placement accuracy compared to the open technique [22–25].

Some studies have shown no significant difference in breach rate between open and percutaneous approach [26, 27]; whereas, Chapman et al, on studying 1609 screws, found a significantly lower breach rate on using the percutaneous technique compared to the open technique [28]. Similarly, our study also showed a significantly lower pedicle breach rate using the percutaneous technique (1.9%) over the open technique (7.9%). Besides the known benefits of minimally invasive techniques such as reduced bleeding risk, damage to soft tissues, and post-operative pain, the improved accuracy of pedicle screw placement suggests that it is a good alternative to the open technique [29]. However, this may not be related to the technique but could be a result of more frequent use of fluoroscopy during minimally invasive posterior stabilization.

From our study, it was also evident that breaches occurred more frequently in the thoracic pedicles than the lumbar pedicles. This could be attributed to the smaller size of the thoracic pedicles and its proximity to the midline when compared to lumbar pedicles [30–34]. Similarly, Parker et al, in their study of 6816 pedicle screws, reported that breaches occur more frequently in the thoracic pedicles than in the lumbar pedicles [11]. However, unlike our cohort, they reported more lateral breaches than medial or superior breaches. Given the higher incidence of breach in thoracic pedicles, we could selectively utilize additional fluoroscopic guidance while screw insertion to avoid screw malpositioning, identify misplaced screws and reposition them intraoperatively.

A cadaveric study done by Agarwal et al found the sensitivity and specificity of postoperative CT scans to pick up pedicle screw breach was 91.52% and 95.02% respectively [35]. However, not all patients with pedicle breaches present with neurological deficits [36–39]. Lotfinia et al reported that nerve root injury with radicular pain and/or neurological deficits was observed only in 15.1% patients with mal-positioned screws [12]. In our study, none of the patients with pedicle screw breach developed neurological deficits. This shows that post-operative CT scans may be too sensitive in picking up pedicle screw breaches that are not of any clinical concern; however, it gave us the opportunity to reassess the intraoperative fluoroscopic images to retrospectively determine the signs of pedicle breach.

With the advent of neuromonitoring, intra-operative assessment of potential pedicle breaches that can compromise neurological integrity has improved [10, 40, 41]. In combination with fluoroscopy, neuromonitoring has become a valuable tool to identify such breaches [42]. However, we did not use neuromonitoring as a routine for trauma patients at our institute during the period of this study as most trauma surgeries were done as emergency procedures. This may have influenced our rate of pedicle breaches. Moreover, being a retrospective study, there are several limitations to be considered. Firstly, we only included consecutive trauma patients who underwent posterior stabilization and we excluded other patients who underwent surgery for indications such as degeneration, deformity, infection and tumours. Also, since the mean age of our selected sample was only 40 years, older patients with degenerative changes having abnormal pedicle entry points or anatomy only constituted a small part our cohort. Considering these factors, our study lacks in overall estimation of pedicle breach at large. In addition, it should also be noted that the surgeries were performed by the same surgical team and our breach rates may not represent that of a surgeon who has newly adopted the technique.

## 5. Conclusion

Among a total of 513 pedicle screws, 28 screws (5.5%) were found to have breached the pedicle; of which, there were 14 minor (<3 mm) and 14 major (≥3 mm) breaches. The minimally invasive technique had a significantly lower breach rate compared to open surgery (1.9% vs. 7.9%). Breaches occurred more frequently in the thoracic pedicles than the lumbar pedicles. We identified certain parameters on intra-operative fluoroscopic imaging to predict a possible pedicle breach: 1) When the length of the pedicle screw spans only up to 50% of the vertebral body but the pedicle screw tip has already transgressed the midline as seen on an AP view, then there is most likely a medial pedicle breach and 2) When the tip of the pedicle screw is overlapped by the screw head or is only minimally visualized on an AP view, then there could be a lateral breach.

## Figures and Tables

**Table 1:** Number of pedicle breaches according to levels.

Level	No. of Pedicle Breaches
Upper Thoracic (T1 – T4)	2
Mid Thoracic (T5 – T8)	1
Lower Thoracic (T9 – T12)	15
Upper Lumbar (L1 – L3)	9
Lower Lumbar (L4 – L5)	1
